# Task shifting of frontline community health workers for cardiovascular risk reduction: design and rationale of a cluster randomised controlled trial (DISHA study) in India

**DOI:** 10.1186/s12889-016-2891-6

**Published:** 2016-03-15

**Authors:** Panniyammakal Jeemon, Gitanjali Narayanan, Dimple Kondal, Kashvi Kahol, Ashok Bharadwaj, Anil Purty, Prakash Negi, Sulaiman Ladhani, Jyoti Sanghvi, Kuldeep Singh, Deksha Kapoor, Nidhi Sobti, Dorothy Lall, Sathyaprakash Manimunda, Supriya Dwivedi, Gurudyal Toteja, Dorairaj Prabhakaran, On behalf of DISHA study investigators

**Affiliations:** Centre for Control of Chronic Conditions, Public Health Foundation of India, New Delhi, India; Centre for Chronic Disease Control, Sector 44, Plot 47, Gurgaon, Haryana India; Rajendra Prasad Government Medical College, Tanda, Himachal Pradesh India; Pondicherry Institute of Medical Sciences, Puducherry, India; Indira Gandhi Medical College, Shimla, India; Aga Khan Health Services, Mumbai, India; Sri Aurbindo Institute of Medical Sciences, Indore, India; National Centre for Disease Informatics and Research, ICMR, Bangalore, India; Indian Council of Medical Research, New Delhi, India

**Keywords:** Task shifting interventions, Cardio-vascular disease, Low and middle-income countries, India

## Abstract

**Background:**

Effective task-shifting interventions targeted at reducing the global cardiovascular disease (CVD) epidemic in low and middle-income countries (LMICs) are urgently needed.

**Methods:**

DISHA is a cluster randomised controlled trial conducted across 10 sites (5 in phase 1 and 5 in phase 2) in India in 120 clusters. At each site, 12 clusters were randomly selected from a district. A cluster is defined as a small village with 250–300 households and well defined geographical boundaries. They were then randomly allocated to intervention and control clusters in a 1:1 allocation sequence. If any of the intervention and control clusters were <10 km apart, one was dropped and replaced with another randomly selected cluster from the same district. The study included a representative baseline cross-sectional survey, development of a structured intervention model, delivery of intervention for a minimum period of 18 months by trained frontline health workers (mainly Anganwadi workers and ASHA workers) and a post intervention survey in a representative sample. The study staff had no information on intervention allocation until the completion of the baseline survey. In order to ensure comparability of data across sites, the DISHA study follows a common protocol and manual of operation with standardized measurement techniques.

**Discussion:**

Our study is the largest community based cluster randomised trial in low and middle-income country settings designed to test the effectiveness of ‘task shifting’ interventions involving frontline health workers for cardiovascular risk reduction.

**Trial registration:**

CTRI/2013/10/004049. Registered 7 October 2013.

**Electronic supplementary material:**

The online version of this article (doi:10.1186/s12889-016-2891-6) contains supplementary material, which is available to authorized users.

## Background

Globally, cardiovascular disease (CVD) is the leading cause of death and disability [1]. Among different regions of the world, low and middle-income countries (LMIC) contribute a disproportionately high burden of CVD (more than two third of the total burden) [2]. As per the recent Global Burden of Disease (GBD) study estimates, one of four deaths in India is attributable to CVD and the epidemic in India is rapidly advancing [3]. The age standardized CVD mortality rates in India is currently well above the global average and that of high income countries [2]. Formulation and effective implementation of evidence-based policies with emphasis on prevention, early detection, and treatment using both conventional and innovative techniques are required to address this major public health problem.

Nearly a quarter of total DALYs (75 % from CVD) in India are attributable to high blood pressure, dietary risk, smoking, high blood sugar and elevated total cholesterol levels [3]. All these risk factors are interwoven with each other, linked to lifestyle changes and amenable to interventions. However, effectiveness of behavioural interventions for lifestyle changes at the population level is not tested systematically in the Indian settings.

A considerable body of research in high income countries with well-established health systems indicates that community health workers (CHWs) in the primary health care system are effective in improving chronic disease care and health outcomes [4]. A network of frontline health workers exists at the village level in India and they are known in different names such as community health workers (CHW), community health representatives (CHR), anganwadi workers (AWW), junior public health nurses (JPHN), health visitors, health inspectors, and accredited social health activists (ASHA). They are often trusted members from the same community and recognized widely in their respective villages. However, they have been so far trained and involved in activities related to maternal and child health and infectious disease management. We propose to train the frontline CHWs as community agents to impart lifestyle changes for cardiovascular risk reduction at the population level (task shifting). A cluster randomised controlled trial (RCT: DISHA study) was designed to test the effectiveness of this approach at the population level. The details of the cluster RCT are summarized below.

## Methods

### Study design and settings

**Fig. 1:**
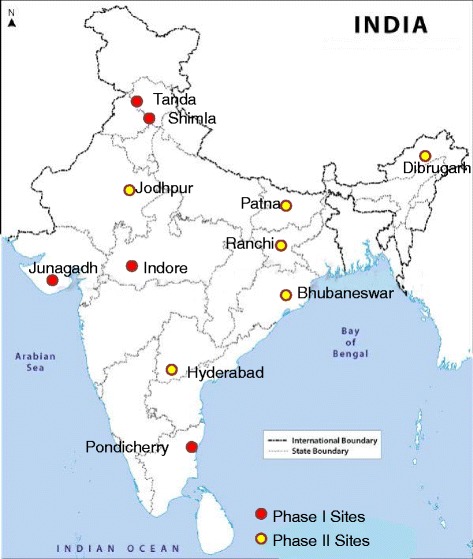
Geographic location of study sites

**Fig. 2:**
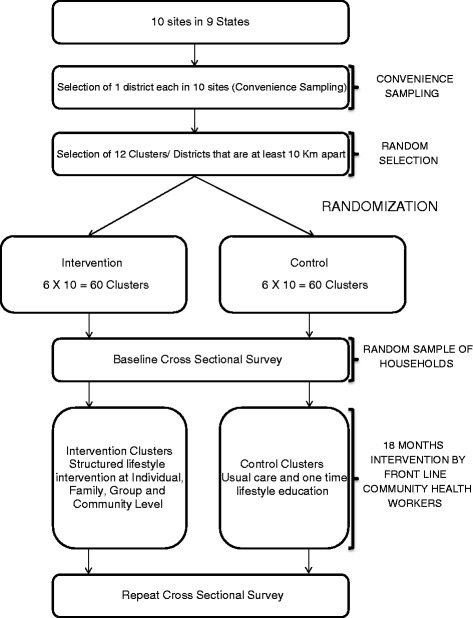
Study flow chart

DISHA is a cluster RCT conducted across 10 sites in India (Fig. 1 and Table 1). At each site, one district was selected based on convenience and from each district 12 clusters were randomly selected using computer generated random numbers (total = 120 clusters in ten sites). A cluster is defined as a small village (ideally including 1–3 Anganwadi Centres) with 250–300 households. The Anganwadi centre is part of the integrated child development service program of Government of India at the village level and typically provides basic health-care including contraceptive counselling and supply, nutrition education and supplementation, and pre-school activities. Over a million such centres are currently active in India. The identified 12 clusters were then randomly allocated to intervention and control clusters in a 1:1 allocation sequence using computer generated random numbers. Random allocation of the clusters was done by a statistician who is unaware of the study settings and cluster locations. If any of the intervention and control clusters were <10 km apart, one was dropped and replaced with another randomly selected cluster from the same district. The iteration continued till we managed to get 6 intervention and 6 control clusters that are at least 10 km apart in each site. The study was initiated in 5 sites in the first phase and in the second phase it has been extended to 5 more additional sites in India. The DISHA study included a baseline cross-sectional survey in all sites involving both the intervention and control clusters with equal representation. The study staff had no information on intervention allocation until the completion of the baseline survey. After completion of the baseline survey in all clusters, frontline health workers (mainly Anganwadi workers and ASHA workers) were trained to deliver CVD risk reduction interventions in the entire intervention clusters for a period of minimum 18 months. A post intervention survey is planned in a representative sample from the same clusters immediately after the intervention phase. The detailed study flow-chart is presented in Fig. 2.

**Table 1:** Characteristics of study sites

Site Name	District/Area	Participating Centre/Medical Institution	Setting	Details
Indore^a^	Sardarpur, Dhar	Sri Aurobindo Institute of Medical Sciences, Indore	Tribal	Predominantly tribal population, and agrarian society. Very poor connectivity by road.
Junagadh^a^	Junagadh, Gujrat	Aga Khan Health Services, Mumbai	Rural	Primarily rural with conservative social practices.
Pondicherry^a^	Pondicherry, Puducherry	Pondicherry Institute of Medical Sciences, Puducherry	Urban	Urban and coastal site with fairly good public health infrastructure available.
Shimla^a^	Mashobra, Himachal Pradesh	Indira Gandhi Medical College, Shimla	Rural	A predominantly agrarian population in a hilly terrain; sparsely populated villages.
Tanda^a^	Bharmour, Himachal Pradesh	Dr Rajendra Prasad Government Medical College, Tanda	Tribal	Predominantly tribal with agriculture as primary occupation.
Bhubaneswar	Kalahandi, Bhawanipatna	Regional Medical Research Centres, Bhubaneswar	Tribal	Predominantly tribal population with major tribes of Kondhas and Souras. The primary occupation is agriculture
	Utnoor, Adilabad	National Institute of Nutrition, Hyderabad	Tribal	Predominantly tribal dominated population. The main occupation is agriculture
Jodhpur	Kotra	Desert Medicines Research Centre, Jodhpur	Tribal	Primarily tribal site, with hilly terrain. The main occupation is agriculture
Ranchi & Patna	Ratu & Namkum	Rajendra Memorial Research Institute of Medical Sciences, Patna (in collaboration with Rajendra Institute of Medical Sciences , Ranchi)	Tribal	Primarily tribal population with agriculture as main occupation.
Dibrugarh	Dibrugarh	Regional Medical Research Centre, North East Region	Tribal	Predominantly tribal population and mostly tea garden workers.

^a^Phase 1 sites

### Baseline risk factor survey

A baseline risk factor survey was carried out initially in the 5 centres included in the first phase (2013–2014) using a structured questionnaire and it is currently on-going in the centres included in the second phase (2014–2015). The survey was conducted among participants from randomly identified 120–150 households in each cluster with a target to obtain a minimum of 300 participants. To account for non-response rate we randomly selected up to 10 % more households from the same cluster initially and in 5 instances up to 20 % more households. All adults over 18 years of age in the randomly selected households were eligible to be included in the survey. The survey questionnaire was translated into local languages (Hindi, Gujarati, Tamil, Oriya, Assamese and Telugu), back translated into English and pilot tested in 30–50 participants at each site (Additional file 1) for internal validity. The survey included assessments of demographic details, general health status, diet, physical activity, tobacco, and alcohol consumption (Table 2). The world health Organization (WHO) STEPS instrument for chronic risk factor surveillance has been modified and adapted to capture local, contextual information. Other components have been adapted from the General Health Questionnaire-12, Fagerstrom Test for Nicotine Dependence (FTND), and WHO AUDIT (Alcohol Use Disorders Identification Test). The general health questionnaire incorporated in the main questionnaire was a measure of current mental health or psychological wellbeing and it is extensively used in different settings and different cultures. A detailed dietary survey was conducted in a sub-sample of 100 random participants at each site. It included, a standardized, semi- quantitative food frequency questionnaire based assessments and two separate 24 h diet recalls within a week time.

**Table 2:** Components of the questionnaire survey

Survey Component	Measures	Source
Socio-demographic details of participant’s family	Age, Sex, Marital Status, Religion, Education, Income, Occupation	CARRS Surveillance Study (2012) [25]
Tobacco Consumption	History, frequency and quantity of tobacco consumption, type of products used	Adapted from Fagerstrom Test for Nicotine Dependence- Indian Adaptation [26, 27]
Alcohol Consumption	History, frequency and quantity of tobacco consumption, type of products used	Adapted from WHO AUDIT [28]
Physical Activity	Levels of sedentary, moderate and vigorous activity levels & time spent doing activity	Adapted from WHO [28] Steps [29]
Knowledge, Attitude & Practices about Blood Pressure	Currently knowledge & attitude toward high blood pressure	Specifically developed for study
Diet and Nutrition	Food Habits and Consumption	Few items adapted from WHO Steps [29] Few items specifically developed for purpose of the study
Medical History	Hypertension/Diabetes/Diabetes – related complications/Hyperlipidemia/Heart Disease	CARRS Surveillance Study (2012) [25]
Family History	Mortality and/morbidity among participant’s family due to cardio-metabolic illness/risk factors	CARRS Surveillance Study (2012) [25]
Treatment History & Expenditure	Expenses for inpatient & outpatient treatment in last 12 months and current	Specifically developed
Medications	Name, dosage and disorder for which medication taken	Specifically developed
Mental Health	General well-being & distress	Adapted in Indian settings [30]

#### Anthropometric and blood pressure measurements

Anthropometric measurements including height (in cm), weight (in kg) and waist circumference (in Inches) was also measured using standardized techniques (Table 3). All measurements were taken with the participant wearing light clothes and the reading were rounded off to one decimal point. Blood pressure (BP) and pulse rate were measured using electronic BP monitors (OMRON 7080). Three measurements were taken, two minutes apart and after resting the participant for at least five minutes before starting the measurement. Participants were instructed not to consume any beverages (coffee, tea or soft drinks) and alcohol at least one hour before taking the measurements. They were also instructed to abstain from smoking. The average of last two BP readings was used for analyses.

**Table 3:** Details of anthropometric measurements

Measurement	Instrument
Height	Stadiometer (Seca)
Weight	Digital weighing scales (Seca)
Waist and hip circumference	Non elastic measuring tapes (Seca)
Anthropometry	Tanita BC-601 segmental body composition analyser, WHO Steps Protocol
Blood pressure measurement	Electronic BP monitor (OMRON 7080) Instrument validated by International Protocol for device validation O’Brien et al., (Working Group on Blood Pressure Monitoring of the European Society of Hypertension)

#### Blood sample collection

In fasting stage, 5 ml of blood was collected, centrifuged at the site for serum and plasma separation after spotting of blood on filter paper, transported and locally stored at the respective laboratory facilities in each site under -20 °C freezers. Spotted filter paper, plasma (1 aliquot) and serum samples (2 aliquots) were transported to the central laboratory at the Indian Council of Medical Research (ICMR), New Delhi following World Health Organization (WHO) guidelines for packaging and transportation of samples. They were then stored at -70 °C freezers. All materials used in sample collection were destroyed using appropriate color-coded bins/bags following the WHO guidelines.

#### Laboratory measurements

The biochemical analyses (Table 4) including lipid profile (total cholesterol, LDL cholesterol and HDL cholesterol), fasting plasma glucose, and haemoglobin were conducted at the central laboratory in batches of samples to ensure standardisation across study sites. The ICMR central laboratory is accredited by the National Accreditation Board for testing and calibration of Laboratories (NABL), India.

**Table 4:** Laboratory measurements

Clinical Parameter	Laboratory parameter	Method
Diabetes	Plasma glucose	Enzymatic Colorimetric Assay method (modified GOD-PAP method based on the work of Trinder, 1969)
Dyslipidemia	Cholesterol	Enzymatic In vitro Calorimetric method (automated clinical chemistry analyzer Roche/Hitachi 902)
	Triglycerides	Enzymatic Calorimetric test (Based on the work by Wahlefeld using lipoprotein lipase from microorganisms for rapid and complete hydrolysis of triglycerides to glycerol followed by oxidation to dihydroxyacetone phosphate and hydrogen peroxide
	High density lipoprotein cholesterol (HDL)	Automated method for direct determination
	Triglycerides	Estimation using Friedewald and Fredrickson Formula, 1972
	Low density lipoprotein cholesterol (LDL)	Homogeneous Enzymatic Assay for direct quantitative determination (automated clinical chemistry analyzer Roche/Hitachi 902), for samples with triglycerides more than 400 mg/dl
	Very low density lipoprotein cholesterol (VLDL)	Estimation using Friedewald and Fredrickson Formula, 1972
Blood Routine	Hemoglobin	Indirect Cyanmethemoglobin method

### Data entry and database

The data entry staff under the supervision of site principal investigator completed data entry on a specially designed web-based online data application. The software has been developed on My SQL 7.0 server. The application uploaded the data directly on a server maintained at the Central Coordinating Centre (CCC), New Delhi. The data are collated, cleaned resolved and analysed at the CCC. We double checked the accuracy of data entry by matching the biochemical data received from the central laboratory with the data entry from the sites. If the data errors were more than 3 %, then we independently verified all other records. We found serious problems in data quality from one of the sites participating in the study and dropped that site from all analyses.

### Intervention development

A package was designed using intervention mapping to guide development of each component of intervention. The following five main steps were involved in the process of intervention mapping; (1) creation of matrices indicating the proximal program objectives from performance objectives and determinants of behaviour and environmental conditions, (2) selection of theory-based intervention methods and practical strategies, (3) design and organising programs, (4) describe adoption and implementation plans, and (5) generate an evaluation plan [5].

In the first step, each component of the healthy lifestyle behaviour related to CVD risk reduction (diet, physical activity, tobacco, alcohol and adherence to treatment) was broken down into performance objectives. They were then prioritized based on practical feasibility of implementation, resource constraints and the local context followed by subjective selection of important and changeable determinants of the risk behaviour. We then framed a matrix of potential change objectives and the corresponding intervention options at individual, group and mass level for population level CVD risk reduction. These objectives were created at each level of intervention planning by crossing performance objectives with determinants and finally deriving the change objectives.

In the next step, the theoretical domains framework was used, as described by Mitchie et al. to guide the development of the potential interventions [6, 7]. A set of 12 domains covering the main factors influencing behaviour and behaviour change were identified: knowledge; skills; social/professional role and identity; beliefs about capabilities; beliefs about consequences; motivation and goals; memory, attention and decision processes; environmental context and resources; social influences; emotions; behavioural regulations; and nature of the behaviours. Using this theoretical framework the tools were designed such as the information booklet, calendar, posters, and leaflets.

The target community was actively involved and played an important role in the development of the intervention tools. The possible barriers and facilitators of a healthy lifestyle were identified through focus group discussions and key informant interviews with target community members, frontline health workers, primary care physicians and the respective site investigators. A proof was prepared initially for each tool (booklets, posters, leaflets, banners and table-top calendars), pilot tested to ensure contextual relevance, and then modified them in an iterative process based on inputs from the pilot test both with frontline health workers and target community members. The frequency of use and the specific target groups (for example; men, women, smokers, individuals with hypertension etc.) were also determined based on focus group discussions and pilot test inputs. The whole process was independently repeated at each site to account for differences in the local context, cultural issues, and language differences. The final print materials are available in the Additional file 2. In addition to the print materials, a calibrated salt spoon and an oil dispenser was also developed based on feedback from the focus group discussions to help each household to measure and quantify their daily consumption of salt and oil.

In the final step a structured intervention program was developed (Table 5). It includes individual level (for example; individual counselling), household level (for example; household visits by frontline health workers) and community level interventions (for example; display of posters, community level activities and competitions). The intervention program was focussed on; 1) healthy diets including low salt, low fats, low trans-fat and high fibre intake, 2) increasing physical activity of vigorous intensity for at least 30 to 60 min and 3) quitting of tobacco and alcohol. The developed tools were assigned to each task and the frequency of intervention was also prescribed in advance.

**Table 5:** Different levels and corresponding tools of interventions

Level of Intervention	Method	Frequency	Tools
Individual Level	Household visits and one to one counselling of household members	Once every two months (9 visits)	Booklet,18- month calendar, hypertension-specific leaflets, healthy lifestyle-specific leaflets, salt spoon to quantify use of salt, oil dispenser to quantify use of oil.
Group	Group meetings with specific target groups such as men, women, youth, persons with hypertension	Once a month (18 meetings)	Recipe demonstrations, video screenings , street theatre, peer led discussions, competitions
Mass	Display of posters or banners with key messages in public places or at gatherings. Distribution of leaflets.	1 poster changed every 3 months	Posters, banners, leaflets

### Delivery of interventions

#### Common interventions

In both the groups, one-time lifestyle education was offered along with baseline risk factor screening by the project staff. All individuals with established risk factors such as hypertension, diabetes and dyslipidemia were referred to the primary care clinics (often primary health centres, community health centres or the adjacent urban health centres) for follow-up care.

#### Structured intervention program

In the intervention arm, the structured intervention program as described earlier was implemented by frontline community health workers identified from the local villages. They were paid a token honorarium for participation in the study during the intervention phase. All the households in the selected clusters were eligible to be included in the intervention program. Before initiating the interventions, the frontline community health workers underwent a two day training program on common CVD risk factors, strategies to prevent the progression of risk factors, lifestyle interventions and also on the structured intervention package. They were also trained to utilise the intervention tools. The duration of intervention period was 18 months. The frontline health workers were instructed to conduct 9 house visits (once in two months) during the intervention period. The objectives of each household visit was described in detail to all participating frontline health workers during the training program. Additionally, the health workers were asked to demonstrate mock household visits during the training program to ensure that they understood the purpose of the planned visits.

#### Household level interventions

The house-visits were micro-planned with the help of the household map for each cluster. A salt spoon as well as an oil dispenser each was distributed to all households in the intervention area. Additionally, each household received a booklet, leaflets and a table top calendar. During each visit, the frontline health workers encouraged family members especially women in the family to use the provided salt spoon and oil dispenser to quantify the amount of salt and oil consumption. Additionally, the family members were briefed about the importance of lifestyle changes using different intervention tools. The family members were also instructed to record the amount of monthly consumption of salt and oil in a log sheet provided along with a table-top calendar. During the home visits, the frontline health workers encouraged participants with hypertension, diabetes and dyslipidemia to seek treatment, take medication regularly and adhere to the prescribed treatment plan.

#### Community level interventions

Apart from the household visits, the frontline health workers also organised public meeting each month in community settings. During these meetings, they gather 30–40 participants and discussed with them about the risk factors associated with CVD and healthy lifestyle behaviours. A community implementation committee comprised of the study investigators at each site, local medical officer, village leaders and volunteers from local self-help groups was organised in each cluster to enable these public meetings. The behaviours being targeted for change were the following; a) consumption of oils rich in mono-unsaturated fatty acids, and poly unsaturated fatty acids, and reduction of trans-fat consumption, b) increase in dietary fibre consumption, c) decrease in dietary salt consumption, d) increase in physical activity, and e) decrease in consumption of tobacco and alcohol. Additionally, several community activities including competition for children and adults (painting competitions, physical activity demonstrations, cooking competitions and healthy recipe demonstrations) and peer led sessions for tobacco and/or alcohol cessation were conducted at the community level in each intervention cluster to sensitize the community.

#### Monitoring and evaluation of intervention

Every three months the intervention process was evaluated in terms of campaign components and the delivery mechanisms. A formal reporting system was established to communicate the details of community level interventions at each cluster to the coordinating centre. Although the interventions were exclusively implemented by frontline health workers, the DISHA project staff at each site monitored the intervention implementation. They visited at least 20 % of households in the intervention clusters at quarterly intervals and documented the progress of interventions in terms of number of visits made by the frontline health workers, messages delivered, and utilization of study tools. Additionally, a team from the coordinating centre conducted monitoring visits to the sites once in six months to verify the implementation process.

### End-line risk factor survey

Similar to the baseline survey, an independent, representative, cross-sectional end-line survey is planned in both the intervention and control clusters. The end-line survey will follow the same methods as in the baseline survey. However in the end-line survey, a customised tablet computer application is developed for data collection in both the intervention and control clusters.

### Study personnel

This multi-site trial is coordinated by a central coordinating team based at the Centre for Chronic Disease Control, New Delhi, India. The team consists of epidemiologists, biostatistician, nutritionists and behaviour communication experts. Each participating site also has at least two investigators, 6 field staff or field attendants recruited for data collection of baseline and post intervention survey, 1 data entry operator for entry of the questionnaires in the software and 1 lab technician for sample collection and processing. Similarly, each study site engaged 6–12 trained frontline health workers for intervention delivery.

### Ethical oversight

The participants were informed about the study and provided with a detailed information sheet. Written informed consent was also obtained from all study participants. The study was approved by the institutional review boards of the central coordinating centre (the Centre for Chronic Disease Control, New Delhi) and all participating sites. The study protocol is registered with the Clinical Trials Registry of India **(**CTRI/2013/10/004049).

### Sample size calculation and statistical power

The sample size was calculated with the aim to detect an epidemiologically significant difference of 2 mmHg in systolic blood pressure at the population level between the intervention and control arm of the study. This is based on the estimate that, a 2 mmHg decrease in mean blood pressure level at the population level will substantially reduce the incidence of hypertension and future cardiovascular events over a longer period of time [8] and the population approach proposed by Geoffrey Rose as an effective means of prevention in apparently healthy populations [9]. We assumed an intra-class correlation coefficient of 0.002 for blood pressure (based on previous cluster design survey data from India), a type 2 error of 5 % and 90 % power for the study. Based on the aforementioned assumptions and accounting for design effect (1+ [m-1] X ICC, where m is the average cluster size), the sample size was estimated as 1620 in each arm. Accounting for a 10 % drop-out rate, we decided to recruit 300 participants from each cluster, 1800 in each arm of the study and 3600 in each study site.

### Statistical analyses

Summary of continuous variables will be presented as means and standard deviations and compared across intervention and control clusters. In case of skewed data, medians and inter-quartile range will be presented. All categorical variables will be presented as frequencies and percentages. Intra-class correlation coefficients for all important risk factors will be also presented.

After the completion of the study, analyses will be performed using cluster level summaries at each site. Equal weight will be given to each of the six clusters. All analysis will compare the two treatment arms, unless otherwise stated. Binary outcomes will be presented as a difference in proportions. Finally, a meta-analysis will be conducted to estimate the overall effect size.

## Discussion

Our study is the largest community based cluster randomised trial in low and middle-income country settings designed to test the effectiveness of ‘task shifting’ interventions involving frontline CHWs for cardiovascular risk reduction. The DISHA study conducted in two phases has adequate power to detect epidemiologically meaningful population level changes in all cardiovascular risk factors.

In order to ensure comparability of data across sites, the DISHA study follows a common protocol and manual of operation with standardized measurement techniques. Furthermore, additional processes such as centralized training, translation of all tools and materials in local languages, and pilot testing and further modification of tools ensured quality of the collected data. Independent verification of all entries in the data application related to biochemical variables with source data helped the coordinating team to evaluate the accuracy of data entry.

The DISHA study will report the ICCs of cardiovascular risk factors at both the village level and household level in adults. The ICC measures the degree to which responses within the same cluster are correlated to each other and it is a major determinant of sample size and power in cluster randomised trials [10]. Ideally the population within a cluster should be as heterogeneous as possible but there should be homogeneity between cluster summary measures. However, homogeneity among units within clusters (high ICC) may elevate the standard error of the estimates of interest and thereby decreases the power of the study substantially [10]. We will be reporting the ICC for all major cardiovascular risk factors both at the village level and household level. The ICC data from this study will be immensely useful for calculating the sample size and designing future cluster RCTs and studies with cluster sampling method.

Lifestyle based CVD prevention programs that employ behaviour change techniques have been extensively implemented in a variety of settings [10–16]. However, the impact of these interventions are mostly assessed at the individual level. To the best of our knowledge, DISHA is the only study sufficiently powered enough to detect meaningful changes in CVD risk factors at the population level in low and middle-income country settings. Community based interventions for CVD risk reduction in the past, especially in the high income country settings, did not yield expected results due to the following reasons; (a) effectiveness of these interventions were tested when there was a strong secular trend in reduction of CVD at the population level, (b) the ‘dose of the intervention’ was not adequate to reach at the community level, (c) interventions were targeted at specific risk factors or risk behaviours, (d) studies were not adequately powered to detect meaningful differences at the population level, and (e) interventions did not take into account the possible barriers and facilitators of adopting a healthy lifestyle [17]. The DISHA study on the contrary is conducted in India when there is a strong secular trend of increase in CVD risk factors at the population level, incorporated mechanism to ensure fidelity of interventions, comprehensively addresses all CVD risk factors and risk behaviours, adequately powered to detect meaningful differences, resource sensitive and culturally tailored to suit to the local needs of the population. Beneficial effect on blood pressure was observed in a cluster RCT of family based home health education delivered by lay health workers in Karachi, Pakistan [18]. The DISHA study is however unique as it measures population level changes (measurements are planned or conducted in independent representative population sample from each clusters before and after intervention) in blood pressure and other CVD risk factors.

In general, behavioural theory based studies or studies with behavioural change techniques on self-regulation and self-monitoring are considered to be more effective lifestyle interventions [19]. The DISHA study not only follows a behavioural change model but adopts a theoretical framework for intervention mapping, describing performance objectives, identifying practical feasibility of implementation, assessing and addressing barriers to intervention, evaluating and prioritising facilitators of intervention and finally describing the change objectives. A strong focus also has been given to self-regulation and self-monitoring with appropriate materials such as measurement tools for oil and salt, diaries and calendars for self-monitoring etc. at the household level.

‘Task shifting’ interventions have been tested in different settings in low and middle-income countries and early results suggest that they are useful in managing non-communicable diseases [20, 21]. Most of these ‘task shifting’ interventions in the past were focused around trained nurses in the health system. The DISHA study focuses on frontline CHWs and shifting their role from managing maternal and child health conditions and infectious disease management to predominantly addressing cardiovascular disease risk factors and risk behaviours at the population level. The DISHA study results will directly inform the current National program for prevention and control of cancer, diabetes, cardiovascular disease and stroke in India to consider scale-up of the ‘task shifting’ strategy at the national level. Other studies on effectiveness of CHWs training in screening for global cardiovascular risk assessment in low and middle-income country settings also support task shifting’ interventions [22, 23]. Although there is a lack of clear definitions for roles and expectations of CHWs, re-designation of health care services and appropriate resource allocation may help scaling-up of ‘task shifting’ interventions similar to the DISHA interventions in LMIC settings [24].

## Summary

The DISHA study is a cluster randomised trial in India, designed to test the effectiveness of ‘task shifting’ interventions involving frontline community health workers for cardiovascular risk reduction. The final results from this study will have direct policy relevance in formulating the health system response to the rising burden of cardiovascular diseases in low and middle-income countries.

## Additional files

Additional file 1: Study questionnaire. http://www.ccdcindia.org//Disha_study/DISHA_STUDY_QUESTIONNAIRE.pdf. (DOC 22 kb) Additional file 2: Intervention tools. http://www.ccdcindia.org//Disha_study/DISHA_Phase_II_IEC_Tools.zip. (DOC 22 kb)
