# Supplementary material for: Task shifting of frontline community health workers for cardiovascular risk reduction: design and rationale of a cluster randomised controlled trial (DISHA study) in India
Source: BMC Public Health. 2016 Mar 15;16:264. doi: 10.1186/s12889-016-2891-6 (PMC4791774; doi:10.1186/s12889-016-2891-6)
Supplement: Additional file 1: — Study questionnaire. http://www.ccdcindia.org//Disha_study/DISHA_STUDY_QUESTIONNAIRE.pdf. (DOC 22 kb) [file 12889_2016_2891_MOESM1_ESM.doc]

**Additional file 2: Study questionnaire**

[http://www.ccdcindia.org//Disha_study/DISHA_STUDY_QUESTIONNAIRE.pdf](http://www.ccdcindia.org/Disha_study/DISHA_STUDY_QUESTIONNAIRE.pdf)
